# Supplementary material for: Transcending the slow bimolecular recombination in lead-halide perovskites for electroluminescence
Source: Nat Commun. 2017 Feb 27;8:14558. doi: 10.1038/ncomms14558 (PMC5333353; doi:10.1038/ncomms14558)
Supplement: Supplementary Information — Supplementary Figures, Supplementary Notes, and Supplementary References [file ncomms14558-s1.pdf]

Supplementary Information

Supplementary Note 1: Physical characterization of the perovskite films

The solution-processed self-assembled perovskite multi-quantum wells (MQWs) film is extremely flat and pin-hole free. The SEM images (Supplementary Fig. 1) indicate that the MQWs film inherits the uniform morphology from two-dimensional (2D) perovskite film. The root-mean-square roughness of the MQWs film is determined to be around 2.6 nm with AFM scanning over an area of 20×20 μm^2. This roughness value is slightly larger than the value (1.4 nm) of 2D perovskite film, but is much smaller than the value (18.8 nm) of 3D perovskite film. In solution-processed thin film, the 3D perovskite typically crystallized in micrometer size single crystals, while the MQWs and 2D perovskite crystallized in domains with average lateral size less than 100 nm (Supplementary Figs. 1 and 2). The thickness of the films used for optical characterization is determined to be around 110 nm, 430 nm, and 650 nm for the perovskite 3D, 2D and MQWs films, respectively (Supplementary Fig. 2). The XRD investigation reveals that the spin-coated 2D and MQWs polycrystalline films are highly oriented with the *C*-axis perpendicular to the film surface (Supplementary Fig. 3). Since the linear absorption and photoluminescence (PL) spectra of the perovskite MQWs is shown in Fig. 1c, the linear absorption spectra of the 2D ($(NMA)_2PbI_4$) and 3D ($FAPbI_3$) perovskites are presented in Supplementary Fig. 4 for comparison.

Supplementary Note 2: Dominant nonradiative decay channels in thin lead-halide 2D perovskites

The direct-bandgap lead-halide single layer 2D (n = 1) perovskites are monomolecular layers of [PbI_6] octahedral sandwiched between long organic barrier layers. The band gap of the barrier layer is much larger than that of the lead halide inorganic well layer (by at least 3 eV), and the dielectric constant of the barrier layer (around 2.1) is much smaller than that (around 6.1) of the well layer. As a result, the excitons are tightly confined by these two effects within the well[1-8]. Unlike 3D perovskites, the termination of the crystal will result in dangling bonds. An ideal lead-halide 2D perovskite has natural out-of-plane self-termination without any dangling bonds. Therefore, near 100% luminescence quantum yield (QY) is expected. However, due to the presence of defects and some other unknown reasons, the QY in lead-halide 2D (n = 1) perovskite bulk crystals has typically been very poor[1-8]. Efficiencies of light emitting diodes (LEDs) constructed with monomolecular layer 2D perovskite are extremely low and typically measured at cryogenic temperatures[1,2]. Here, through detailed pump fluence and temperature dependent ultrafast spectroscopy investigation of the 2D $(NMA)_2PbI_4$ perovskite, we show that the injected carriers decay through significant surface trapping, intrinsic defect trapping, exciton-phonon coupling and exciton-exciton interaction. These decay processes can effectively compete with the band edge radiative recombination and are responsible for the low luminescence QY observed.

Supplementary Fig. 5a shows the temperature dependent PL spectra of the $(NMA)_2PbI_4$ following continuous wave laser excitation (1 mWcm^{-2}). At room temperature, no trap-related below band gap emission could be clearly identified. The band edge (BE) PL with a long low energy side tail dominates the light emission. However, when the temperature is reduced, the presence of large population trap states manifests as additional two broad below band gap emission bands. The integrated emission intensity of these bands is comparable with the BE emission intensity at 80K. The trapped exciton emission at room temperature is negligible in $(NMA)_2PbI_4$, possibly due to the low oscillator strength and low QY. The first emission band peaks at 565 nm

with a full width at half maximum (FWHM) of around 25 nm. The second band peaked at 620 nm possesses a FWHM of around 100 nm. The second white light emission band typically is attributed to self-trapped exciton emission in the 2D perovskites, which is caused by the intrinsic crystal lattice defects[9]. Lifetime of this localized exciton recombination (around 15 ns) is much longer than that of the BE exciton recombination (around 250 ps) (Fig. 2c and Supplementary Fig. 5d). The emission intensity of this broad band could also be tailored over a range of wavelengths by varying the length of the organic barrier layer. For the first band (565 nm), Time-resolved PL (TRPL) trace in Supplementary Fig. 6 around this peak shows a building-up time of around 160 ps, which is closely matched with a fast decay time of the BE luminescence. This result indicates that carrier localization from band edge to this emission band related states requires a relatively long time delay (around 160 ps). Since this carrier localization time is close to the charge transfer time across the thin film interface, we attribute it to surface states (SS) related emission[9,10]. Supplementary Fig. 5b also shows that both the BE emission intensity and emission peak energy increase with decreasing the temperature. The temperature dependence of the BE PL peak energy could be well-fitted by the Bose-Einstein equation with extracted band gap of 2.4 eV at 0 K and 2.38 eV at 300 K[11]. The temperature dependence of the BE emission intensity could be described by an Arrhenius plot with two thermal activation processes, with activation energies of 488 meV and 76 meV, respectively[11]. Due to the thermal-activated exciton trapping and large trap states density, Supplementary Fig. 5b clearly shows that the BE emission intensity decrease over 80% when the temperature is increased from 80 K to 300 K.

With intensified fs laser pulses excitation, Supplementary Fig. 5c shows that there is another sharp peak (525 nm) emerges at the low energy side of the BE peak (517 nm) at 80 K. The intensity ratio of this peak to BE peak increases with increasing the pump fluence. Considering the 2D perovskite's characteristics, free electrons and holes tend to irreversibly form excitons due to the large exciton binding energy (around 300 meV). At high injected carrier density, stable biexcitons are also expected to exist due to the large exciton binding energy. Therefore, the high power-induced sharp emission peak (Supplementary Fig. 5c) could be reasonably attributed to radiative decay of a biexciton to a transverse exciton. The emission QY should be quite low as this process involves multi-particle interactions and not through a direct gap two particle recombination. The biexciton binding energy, that is the energy difference between the biexciton emission peak and single exciton emission peak, is determined to be around 37 meV. This value is much larger than the biexciton binding energy in conventional semiconductors (typical few meV or less)[12], and is also much larger than the room temperature thermal activation energy (around 25 meV). The biexciton recombination should be a significant decay channel at high injected carrier densities in 2D lead-halide perovskites.

In additional to the carrier trapping and exciton-exciton interaction, exciton-phonon interaction is another efficient energy loss channel in 2D lead-halide perovskite bulk crystals. The 2D perovskites are few layers of [PbI_6] octahedral sandwiched between long organic barrier layers. The self-assembled QWs are weakly bounded together with van der Waals interaction in contrast to inorganic 3D semiconductors bounded with covalent bonds. Hence, the excess charge will cause large perturbation of the lattice and is coupled with the lattice phonons. To examine the exciton-phonon coupling strength in 2D perovskite, the BE emission broadening with temperature was investigated. Previously, at low injected carrier density, this broadening is believed to primarily arise from exciton-phonon interactions. The FWHM of the emission spectra is expressed as[6,11]:

$$\Gamma = \Gamma_{\mathrm{inh}} + \gamma_{\mathrm{LA}} T + \frac{\Gamma_{\mathrm{LO}}}{\exp(\hbar\omega_{\mathrm{LO}}/k_{\mathrm{B}}T)-1} \qquad \text{(Supplementary equation 1)}$$

where Γ_{inh} is the inhomogeneous broadening factor, γ_{LA} is exciton- longitudinal acoustic (LA) phonon coupling strength, Γ_{LO} is exciton- longitudinal optical (LO) phonon coupling strength, and $\hbar\omega_{\text{LO}}$ is the LO phonon energy. Supplementary Fig. 7 shows that the experimental result can be well-described with this phenomenological model. The fitted parameters are Γ_{inh}= 25 ± 5 meV, γ_{LA}= 0.07 ± 1 meVK^{-1}, $\hbar\omega_{\text{LO}}$= 60 ± 10 meV and Γ_{LO} = 260 ± 50 meV. In comparison, the exciton-LO (exciton-LA) phonon coupling strength is reported to be in a range of 9.5-63.6 meV (0.06-1.96×10^{-3} meVK^{-1}) for most of the 3D inorganic semiconductors (CdS, CdSe, CdTe, ZnTe, ZnSe, GaAs, InP)[13]; around 86 ± 18 meV (0.042 ± 0.011 meVK^{-1}) for monolayer MoS_2[14]; and in a range of 40-92 meV for 3D perovskites ($MAPbI_3$, $MAPbBr_3$, $FAPbI_3$, and $FAPbBr_3$,)[15,16]. Both the exciton-LA phonon and exciton-LO phonon coupling strengths in lead-halide 2D perovskite are noticeably larger.

With strong exciton-phonon interaction, the light emission could occur at an energy which is much smaller than free exciton energy by coupling part of the exciton energy to phonons. Therefore, the strong exciton-phonon interaction will manifest as a long low energy luminescence tail, which is clearly observed in both steady state PL spectra (Supplementary Fig. 5a) and TRPL image (Supplementary Fig. 8a) of $(NMA)_2PbI_4$ at room temperature. For comparison, the TRPL image of 3D $FAPbI_3$ is shown in Supplementary Fig. 8b. The low energy luminescence tail is not obvious for 3D perovskite at similar injected carrier density and pump photon energy. These results indicate that the charge-phonon interaction in 2D perovskite bulk crystal is much strong than that in 3D perovskite. The corner-sharing lead halide octahedral (PbX_6^{4-}) 3D crystal is less affected by excess charges. The low energy luminescence tail can be summarized by Maxwell-Boltzmann distribution with an effective phonon temperature (T_{phonon}) as[17]:

$$I(h\nu) = Aexp(-(h\nu_0 - h\nu)/K_{\text{B}}T_{\text{phonon}}) \quad \text{(Supplementary equation 2)}$$

where $I(h\nu)$ is the luminescence intensity from 550 nm to 700 nm, $h\nu_0$ is the luminescence peak energy, K_{B} is the Boltzmann constant. T_{phonon} is extracted by fitting the low energy luminescence tail with Supplementary equation 2. The fitted results show that T_{phonon} exponentially increases with increasing the pump fluence from 0.1 μJcm^{-2} to 5 μJcm^{-2}. T_{phonon} reaches a maximum value of around 1780 K at pump fluence over 5 μJcm^{-2} with 3.1 eV photon excitation (Supplementary Figs. 8c and 8d). The high pump fluence induced hot phonons will cool down with a lifetime of around 60 ps, which is independent on the pump fluence (Supplementary Fig. 9).

Supplementary Note 3: Determine the exciton localization efficiency from bilayer QW to TQW in the MQWs film

As discussed in main text, the transient absorption (TA) and TRPL measurements (Figs. 1d, 2 and Supplementary Figs. 10-12) clearly show that exciton localization from thin (inorganic layer number $n \leq 4$) QWs to thick (T) ($n \geq 5$) QWs is extremely fast with a transfer time of around 0.5 ps and a relative slow transfer time of around 200 ps. These fast exciton localization times suggest that exciton localization efficiency from thin QWs to TQWs in the MQWs film should be very high. To extract the detailed exciton localization efficiency, we modeled pump fluence dependent light emission at bilayer resonance in the MQWs film. Under fs laser pulse excitation and the assumption that exciton recombination in TQWs is much slower than that in bilayer QWs, the dynamics of photo-generated exciton density (n_2) in bilayer QWs can be described with the following set of differential equations:

$$\frac{dn_2(t)}{dt} = -a_f n_2(t) n_{TQWs}^f(t) - a_s n_2(t) n_{TQWs}^s(t) - bn_2(t) - cn_2^2(t) \quad \text{(Supplementary equation 3)}$$

$$\frac{dn_{TQWs}^f(t)}{dt} = -a_f n_2(t) n_{TQWs}^f(t) \quad \text{(Supplementary equation 4)}$$

$$\frac{dn_{TQWs}^s(t)}{dt} = -a_s n_2(t) n_{TQWs}^s(t) \quad \text{(Supplementary equation 5)}$$

where $n_{TQWs}^f(t)$ and $n_{TQWs}^s(t)$ are the bilayer QWs closely attached and non-closely attached TQWs state densities, respectively. The a (a_f, a_s) is the corresponding product of localization cross section and carrier velocity. The b is the exciton recombination constant in bilayer QW. The c is the bi-exciton Auger recombination constant in bilayer QW. Therefore the first two terms in Supplementary equation 3 represent the fast and slow exciton localization pathways, the third term denotes the radiative recombination at the bilayer QW resonance, while the fourth term is biexcitonic Auger recombination. Thus the relationship between the integrated bilayer exciton luminescence intensity ($I_{PL} = k\int_0^\infty b \cdot n_2(t)dt \sim kbn_2(0)\tau_2^e$, where k is a constant, τ_2^e is the effective lifetime of bilayer exciton) and the initial photogenerated exciton density $n_2(0)$ can be obtained as:

$$n_2(0) = n_{TQWs}^f(0)(1 - e^{-a_f I_{PL}/kb}) + n_{TQWs}^s(0)(1 - e^{-a_s I_{PL}/kb}) + I_{PL}/k + cn_2(0)I_{PL}/(2kb)$$
(Supplementary equation 6)

The experimental result can be well-fitted with Supplementary equation 6 (inset of Supplementary Fig. 11d). Based on the fitted parameters, the exciton localization efficiency could be calculated with $(n_{TQWs}^f(0)(1 - e^{-a_f I_{PL}/kb}) + n_{TQWs}^s(0)(1 - e^{-a_s I_{PL}/kb}))/n_2(0)$. The injected carrier density dependent exciton localization efficiency is extracted and presented in Supplementary Fig. 11d. Since we neglect the higher order multiparticle Auger recombination, the exciton localization efficiency could be overestimated at injected carrier density higher than 10^{17} cm^{-3}. However, at carrier densities lower than 10^{16} cm^{-3}, where the Auger recombination is negligible, the exciton localization efficiency is estimated to be over 85% (Supplementary Fig. 11d).

With the abovementioned dynamical exciton model, the dependence of the bilayer QW PL effective lifetime on injected exciton density also can be well-described with the following equation (Fig. 2c):

$$\tau_2^e = \frac{n_2(0)\tau_2^e - c(n_2(0)\tau_2^e)^2/2}{n_{TQWs}^f(0)(1 - e^{-a_f n_2(0)\tau_2^e}) + n_{TQWs}^s(0)(1 - e^{-a_s n_2(0)\tau_2^e}) + bn_2(0)\tau_2^e} \quad \text{(Supplementary equation 7)}$$

Through fitting, the bilayer biexciton recombination coefficient in QW assembles is extracted to be around $1.3 \times 10^{-20} cm^3 ps^{-1}$. With the reported thickness of 1.272nm[5], the biexciton recombination coefficient of single bilayer QW is estimated to be around $0.1 cm^2 s^{-1}$.

Similarly, the exciton dynamics in pure single layer QW can be described with the exciton recombination coupled with biexciton recombination. Therefore, the PL effective lifetime can be described with:

$$\tau_1^e = \frac{1}{b + c n_1(0)/2} \quad \text{(Supplementary equation 8)}$$

By fitting the experimental result with Supplementary equation 8 (Fig. 2c), the biexciton recombination coefficient in single layer QW assembles is extracted to be around $2.8 \times 10^{-20} cm^3 ps^{-1}$. Considering the reported thickness of 0.636nm[5], the biexciton recombination coefficient of single layer QW is estimated to be around $0.44 cm^2 s^{-1}$. This value is quite close to the reported biexciton recombination coefficient (around $0.5 cm^2 s^{-1}$) for single layer MoS_2[18]. The above results also show that the biexciton recombination coefficient decreases with increasing the QW width (Table 1).

Supplementary Note 4: Determine the carrier recombination coefficients in TQWs and 3D perovskite

In 3D perovskite, the carrier kinetics can be well-described with equation (1). With the approximation that carrier dynamics with an effective lifetime (τ_{3D}^e), the following relation could be obtained:

$$\tau_{3D}^e = \frac{1}{k_1 + k_2 n_{3D}(0)/2 + k_3 n_{3D}^2(0)/3} \quad \text{(Supplementary equation 9)}$$

The dependence of PL effective lifetime on injected carrier density of the 3D perovskite can be well-fitted with the above equation (Fig. 3c). The fitted recombination coefficients are $k_1 = 9(\pm 4) \times 10^5 s^{-1}$, $k_2 = 7(\pm 3) \times 10^{-10} cm^3 s^{-1}$ and $k_3 = 3(\pm 2) \times 10^{-28} cm^6 s^{-1}$ (Table 1). These values are matched with the recombination coefficients obtained from global fitting of the power dependent TRPL dynamics with equation (1) (Supplementary Fig. 13a), which are: $k_1 = 1(\pm 3) \times 10^6 s^{-1}$, $k_2 = 5(\pm 2) \times 10^{-10} cm^3 s^{-1}$ and $k_3 = 4(\pm 3) \times 10^{-27} cm^6 s^{-1}$. The trap associated recombination coefficient k_1 extracted here is nearly one order of magnitude smaller than the typically reported $\sim 1 \times 10^7 s^{-1}$ [10,19-23], which indicates the high quality 3D perovskite produced here. The extracted k_2 and k_3 values are consistent with previously reported $k_2 \sim 1 \times 10^{-10} cm^3 s^{-1}$ and $k_3 \sim 1 \times 10^{-28} cm^6 s^{-1}$ [10,20-23], which are intrinsic properties of the 3D perovskite.

As discussed in the main text, the carrier dynamics in TQWs can be described with exciton recombination coupled with bimolecular recombination and three-particle Auger recombination. Therefore, the carrier dynamics in the TQW assembles could also be described with equation (1). However, the first order recombination is mainly associated with exciton recombination here, not the trap-related recombination as in 3D perovskites. Therefore, the carrier density dependence of PL effective lifetime could also be fitted with Supplementary equation 9 (Fig. 3c). The fitted parameters are $k_1 = 5(\pm 3) \times 10^7 s^{-1}$, $k_2 = 2(\pm 0.6) \times 10^{-9} cm^3 s^{-1}$ and $k_3 = 2(\pm 1) \times 10^{-26} cm^6 s^{-1}$ (Table

1). These values are closely matched with the recombination values ($k_1 = 3(\pm1)\times10^{7} s^{-1}$, $k_2 = 2(\pm1)\times10^{-9} cm^3 s^{-1}$ and $k_3 = 4(\pm2)\times10^{-26} cm^6 s^{-1}$) extracted by global fitting of the pump fluence dependent TRPL decay curves with equation (1) (Supplementary Fig. 13b). Under steady-state electric injection, the radiative emission QY of the TQWs is given by

$$\eta(n) = \frac{k_1 + nk_2}{k_1 + k_{Trap} + nk_2 + n^2 k_3} \qquad \text{(Supplementary equation 10)}$$

With the above extracted carrier recombination coefficients and the assumption that carrier localization from bilayer QWs to TQWs is near unity and similar trap states density associated with MQWs film as in 3D perovskite ($k_{\mathrm{Trap}} \sim 10^6$ s^{-1}), the internal radiative emission QYs of the MQWs film and 3D perovskite are calculated with Supplementary equation 10 and equation (2), respectively. Supplementary Fig. 14 clearly shows that the predicted carrier density dependences of the emission QYs are very close to the experimental observations (Fig. 3d). At injected carrier density below 10^{16} cm^{-3}, the MQWs shows near invariance and much higher radiative emission QY than that of the 3D perovskite. However, due to the three-particle Auger recombination coefficient in MQW film is nearly two orders of magnitude larger than that in 3D perovskite, the radiative emission QY of the MQWs film is lower than that of 3D perovskite at carrier density above 1.5×10^{16} cm^{-3}.

Supplementary Note 5: Determine the trap states density associated with TQWs and 3D perovskite

Supplementary Fig. 15a shows the light soaking induced integrated PL relative intensity change with time for TQWs in the self-assembled MQWs film as well as for 3D perovskite. The greatly increased PL intensity indicates part of the trap states in 3D perovskite were passivated with light induced re-crystallization[24], while the nearly invariant PL intensity indicates that the trap state density in TQWs is negligible. The detailed trap states densities associated with TQWs and 3D perovskite were estimated with injected-carrier density dependent PL measurements[25].

Under low fluence fs laser pulse excitation (where Auger recombination is negligible) and the assumption that trap states recombination is much slower than band edge radiative recombination, the initial photogenerated carrier density $n_C(0)$ can be obtained as:

$$n_c(0) = \sum_i n_{TP}^i(0)(1 - e^{-a_i \tau_0 I_{PL}/k}) + I_{PL}/k \qquad \text{(Supplementary equation 11)}$$

where $n_{TP}^i(t)$ is the trap states density and a_i is the product of the trapping cross section and the carrier velocity. The k is a constant and τ_0 is intrinsic PL lifetime. The first term in Supplementary equation 11 represents the injected carriers consumed through various trap-mediated non-radiative pathways, while the second term denotes carriers decayed through radiative recombination inside the film. Fitting the experimental results with Supplementary equation 11 yields a trap states density of $n_{TP}^{3D} \sim 3.5\times10^{16}$ cm^{-3} for the 3D perovskite (Supplementary Fig. 15b). In comparison, the trap states density associated with the TQWs is estimated to be much lower than that of the 3D perovskite ($n_{TP}^{TQWs} < 3\times10^{13}$ cm^{-3}) (Table 1). This conclusion is also consistent with the above light soaking results.

Supplementary Note 6: Estimate the TQWs doping concentration in the self assembled MQWs film

When carriers are directly injected into the TQWs with selective fs laser pulses excitation, the possibility (*P(n)*) of the TQW domain occupied with *n* electron-hole pairs follows the Poisson distribution: $P(n) = <N>^n e^{-<N>} / n!$, where <N> is the average photo-injected electron-hole pairs per TQW domain. Then the initial time PL intensity ($I_{PL}[t = 0]$) could be expressed as:

$$I_{PL}[t=0] \propto m_0 P(1) k_1 + \sum_{i=2}^{\infty} m_0 P(i) i^2 k_2 = k_2 n(0) + k_2 n^2(0) / m_0 + n(0)(k_1 - k_2) e^{-(n_0 / m_0)}$$

(Supplementary equation 12)

where m_0 is TQWs doping concentration in the self-assembled film, *n(0)* is the photo-injected carrier density, k_1 is the exciton recombination coefficient, k_2 is the electron-hole bimolecular recombination coefficient. Here we assume that the carriers will decay via bimolecular recombination when the injected electron-hole pair number is larger than 1. When a single electron-hole pair is injected into the TQW, the carriers will decay via exciton recombination. Fitting the experimental result with Supplementary equation 12 (Supplementary Fig. 16), the TQWs doping concentration is estimated to be around 9×10^{16} cm^{-3}.

Supplementary Note 7: Examine the light emission uniformity of the MQWs film

With intensified fs UV laser pulses (400 nm) excitation, the photo-stability of the self-assembled MQWs film is similar to that of the 3D $FAPbI_3$ film, and is much better than that of the 2D perovskite film (Supplementary Fig. 17). The light emission uniformity of the films was examined with integrated PL intensity and lifetime mapping over large areas (80×80 μm^2) (Supplementary Fig. 18). The spatial homogeneity shows an integrated PL intensity standard deviation of ±10%, ±20% and ±39% for the MQWs, 2D and 3D perovskite films, respectively. The light emission inhomogeneity of the 2D perovskite film may be overestimated due to the low PL intensity collected. This result indicates excellent homogeneity over large areas for the self-assembled MQWs film, which is also consistent with the relative high TQWs doping concentration (around 9×10^{16} cm^{-3}) estimated above. Otherwise, isolated bright light emission centers (TQWs) could be clearly resolved with the confocal microscopy (around 1 μm spatial resolution). The TQWs distribution over Z-axis in the self-assembled film (thickness around 650 nm) was examined by comparing PL lifetimes collected from front side and back side of the film. Since PL lifetime increases with increasing well width of the perovskite QWs, a large PL lifetime difference between the light emission collected from the front side and the back side is expected if the film with a QW width gradient distribution across the Z-axis. However, Supplementary Fig. 19 clearly shows that the PL lifetime difference is negligible between the light emissions collected from both sides.

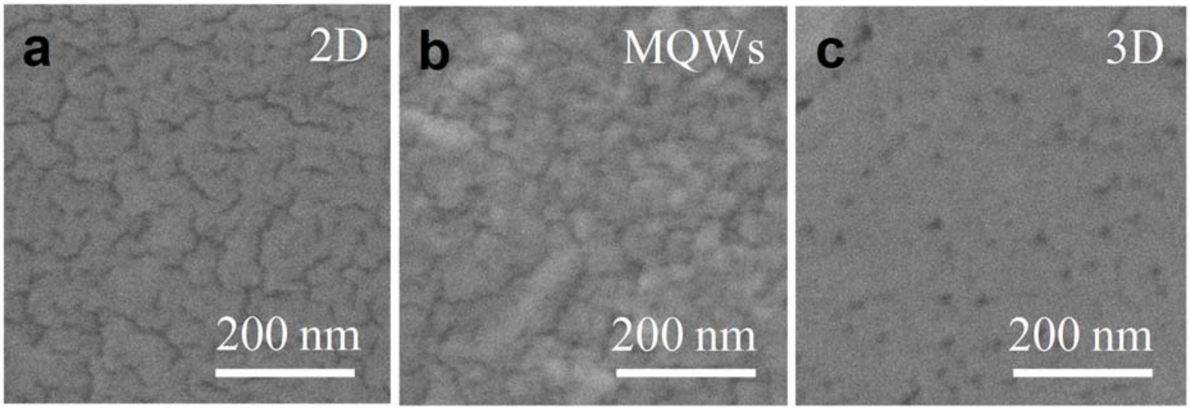

Supplementary Figure 1 | Scanning electron microscope (SEM) Images. SEM images of the two-dimensional (2D), multi-quantum wells (MQWs) and 3D perovskite thin films. Collected at a 3 KeV operating voltage.

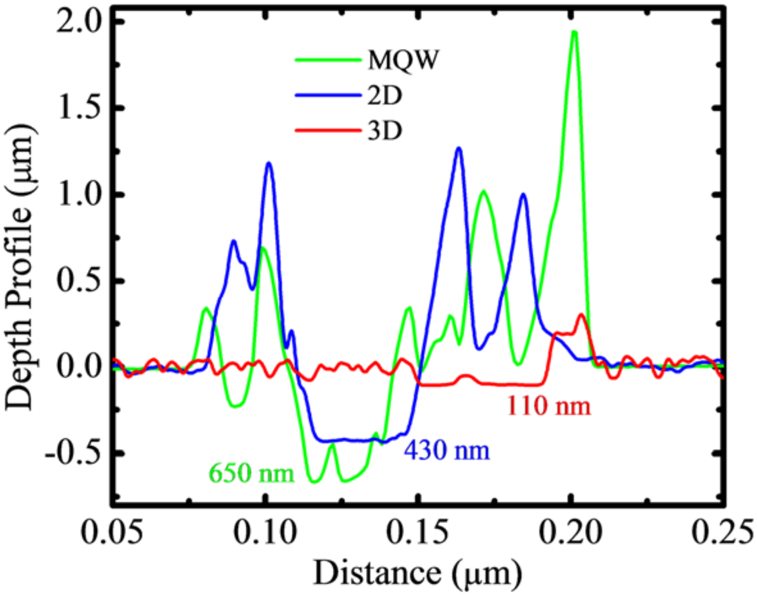

Supplementary Figure 2 | Film thickness determination. The step profiles show the thicknesses of the multi-quantum wells (MQWs) (green), two-dimensional (2D) (blue) and 3D (red) perovskite films used for optical characterization.

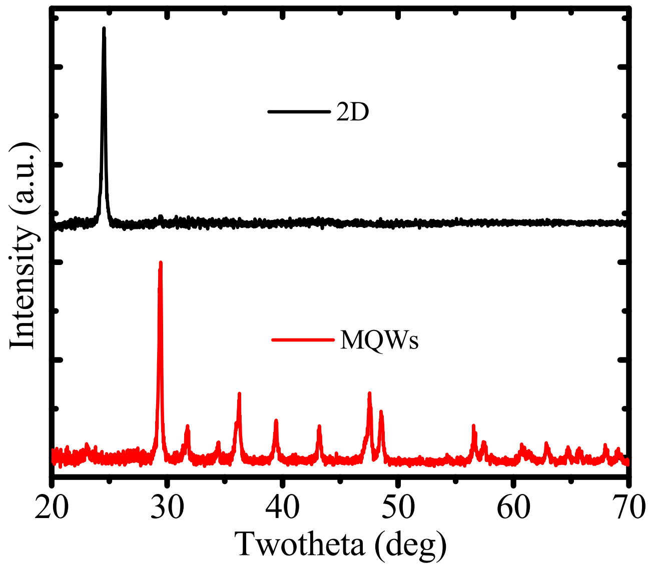

Supplementary Figure 3 | X-ray diffraction (XRD) pattern. XRD pattern of the two-dimensional (2D) and multi-quantum wells (MQWs) thin films.

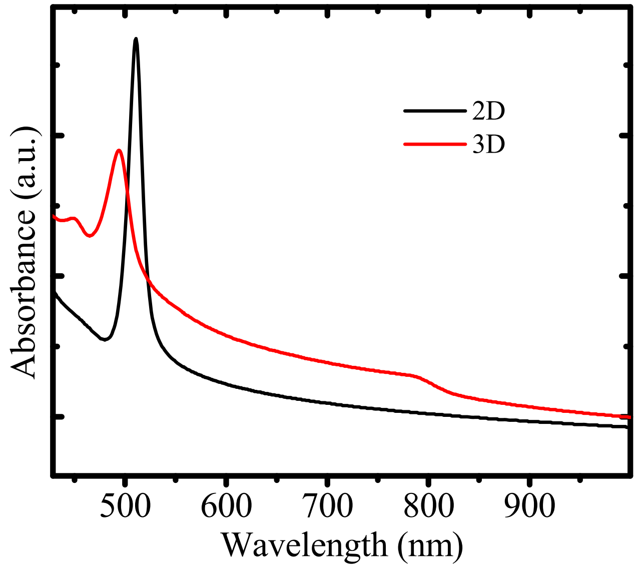

Supplementary Figure 4 | Absorption of the perovskite films. Linear absorption spectra of the two-dimensional (2D) and 3D perovskite thin films.

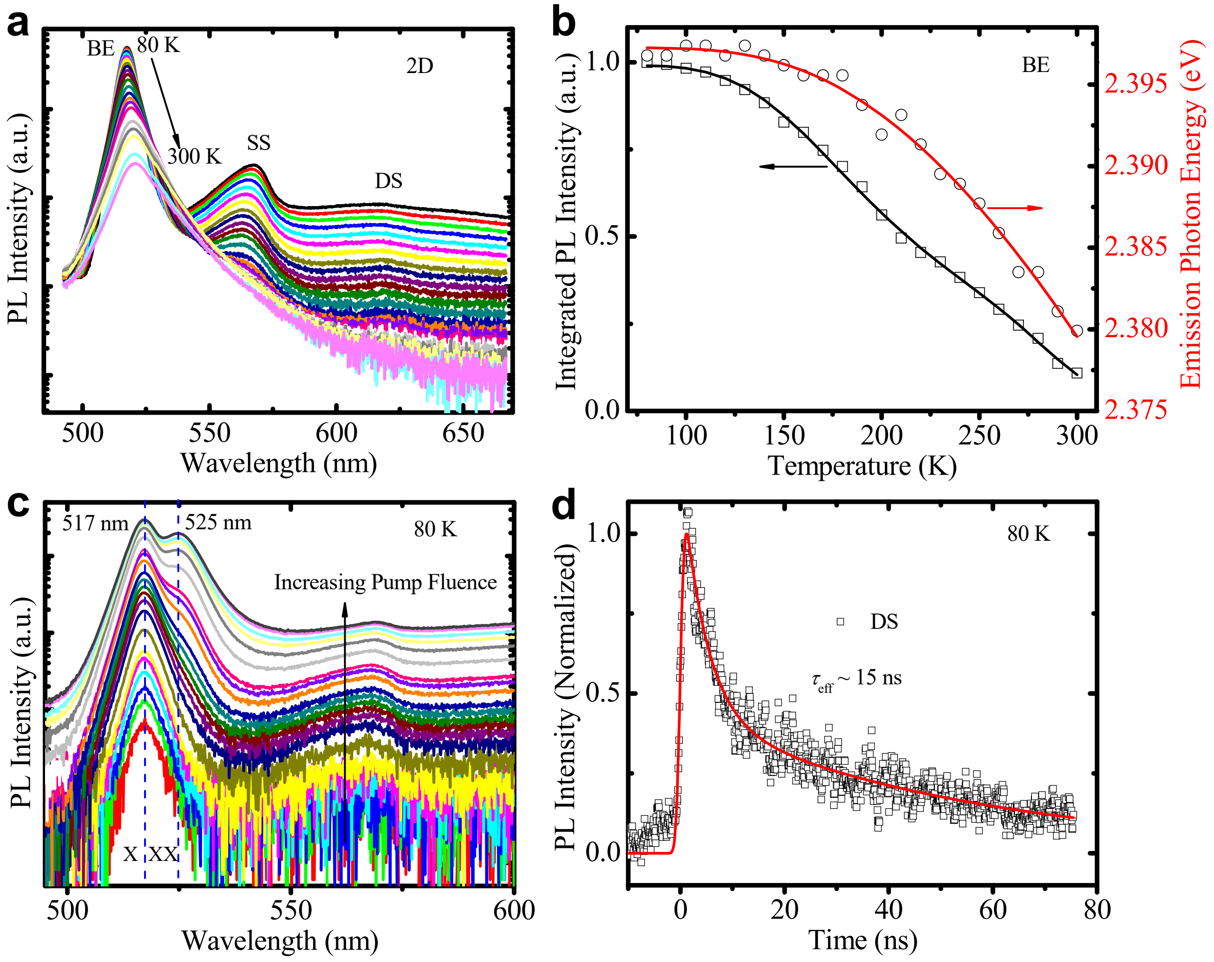

Supplementary Figure 5 | Illustration of the main exciton decay channels in two-dimensional (2D) perovskite. (a) Temperature-dependent photoluminescence (PL) spectra of 2D perovskite $(C_{10}H_7CH_2NH_3)_2PbI_4$ following continuous wave laser excitation at 475 nm (1 mWcm^{-2}). SS represents the surface states (SS) related emission. **(b)** The extracted temperature dependences of the band edge (BE) integrated PL intensity and emission peak photon energy as indicated in **(a)**. The solid lines are fittings as described in the text. **(c)** Pump fluence dependent PL spectra of the 2D perovskite at 80 K following fs laser excitation at 400 nm (50 fs, 1 KHz, 0.001-16 μJcm^{-2}). **(d)** Normalized PL kinetics for the defect states (DS) as indicated in **(a)** following excitation at 400 nm (50 fs, 1 KHz, 0.01 μJcm^{-2}).

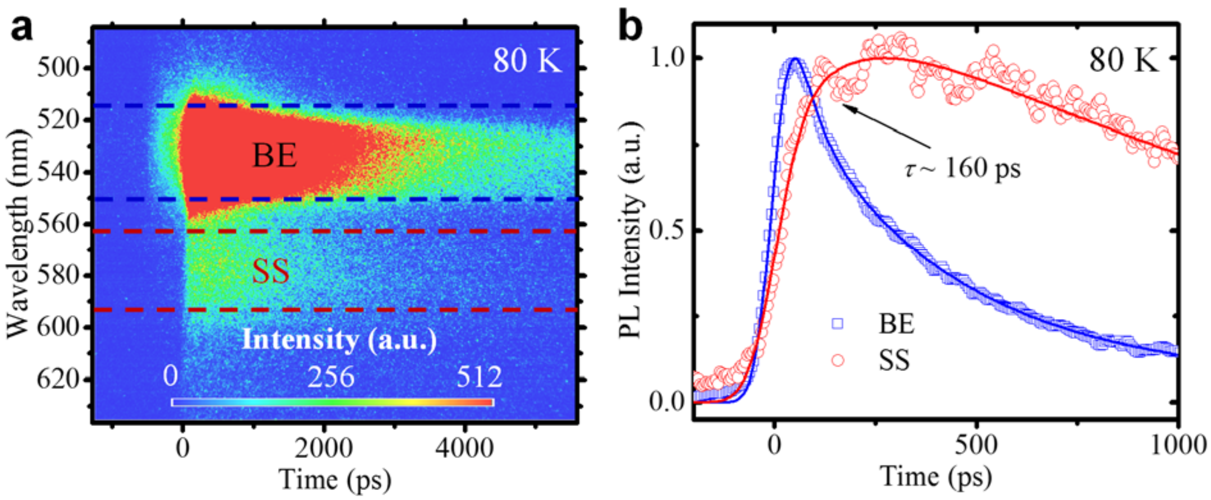

Supplementary Figure 6 | Reveal surface states (SS) emission in the two-dimensional (2D) perovskite. (a) Pseudo color time-resolved photoluminescence (TRPL) plot showing the light emission intensity as a function of emission wavelength and delay time for the 2D $(C_{10}H_7CH_2NH_3)_2PbI_4$ thin film at 80 K. **(b)** Normalized TRPL decay dynamics at band edge (BE) and SS (extracted from **a**) are overlaid for comparison. Following excitation at 400 nm (50 fs, 1 KHz, 0.06 μJcm^{-2}).

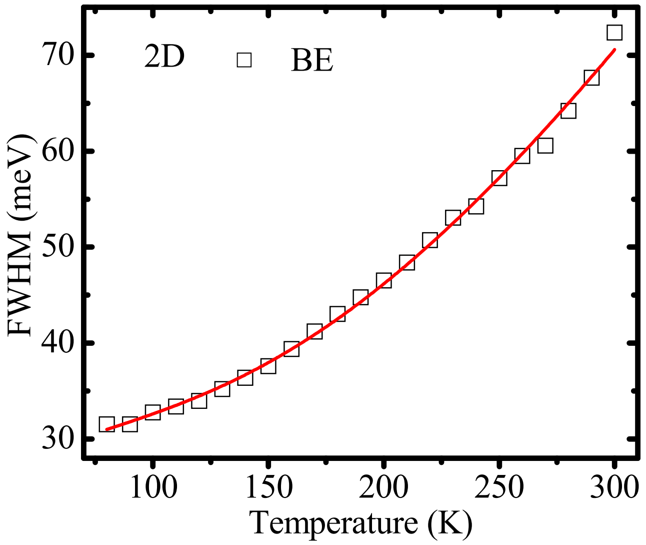

Supplementary Figure 7 | Exciton-phonon coupling strength in the two-dimensional (2D) perovskites. The band edge (BE) emission full width at half maximum (FWHM) as a function of temperature for 2D perovskite $(C_{10}H_7CH_2NH_3)_2PbI_4$ following continuous wave laser excitation at 475 nm (1 mWcm^{-2}). The solid line is the fitting with Supplementary equation 1.

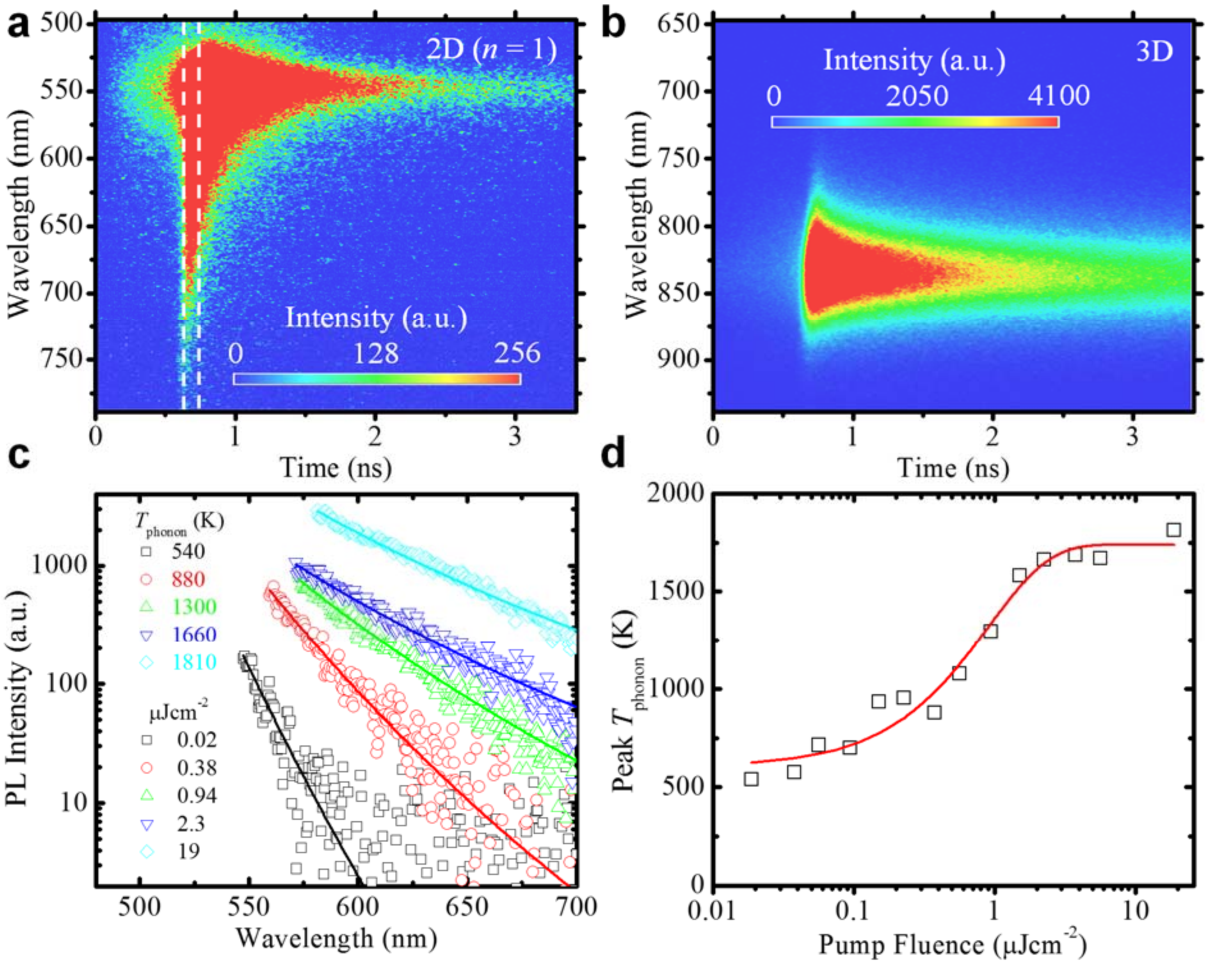

Supplementary Figure 8 | Strong exciton-phonon coupling in the two-dimensional (2D) perovskites. Pseudo color time-resolved photoluminescence (TRPL) plot showing the light emission intensity as a function of emission wavelength and delay time for the 2D $(C_{10}H_7CH_2NH_3)_2PbI_4$ (**a**) and 3D $FAPbI_3$ (**b**) perovskite thin films at room temperature. The strong exciton-phonon coupling induced low energy side broadening of band edge luminescence is clearly revealed in the 2D perovskite. The samples were excited with 400 nm laser pulses (50 fs, 1 KHz, around 5 μJcm^{-2}). (**c**) The low energy side broadening as a function of pump fluence for the 2D perovskite at the initial time as indicated in (**a**). The low energy side tails are fitted by Maxwell-Boltzmann distribution as described in Supplementary equation 2 to extract the hot phonon effective temperature (T_{phonon}). (**d**) The extracted T_{phonon} as a function of pump fluence.

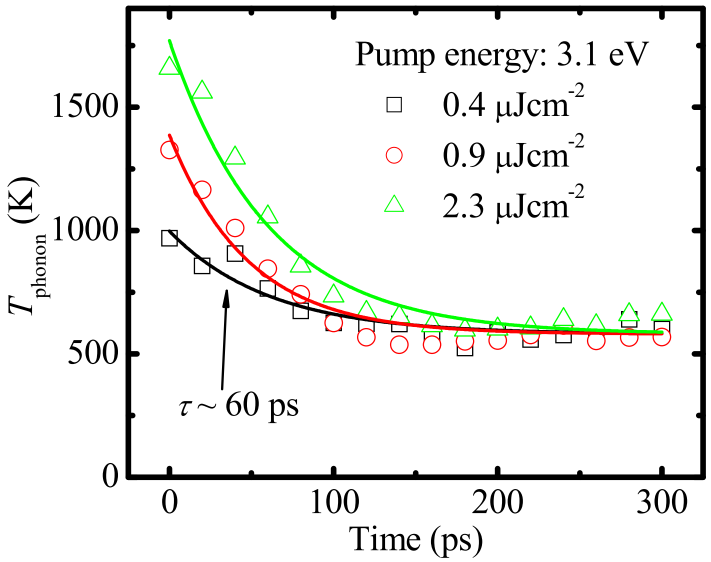

Supplementary Figure 9 | Hot phonon decay in the two-dimensional (2D) perovskite. Time-dependent T_{phonon} at different pump fluence. The solid lines are single exponential decay fittings.

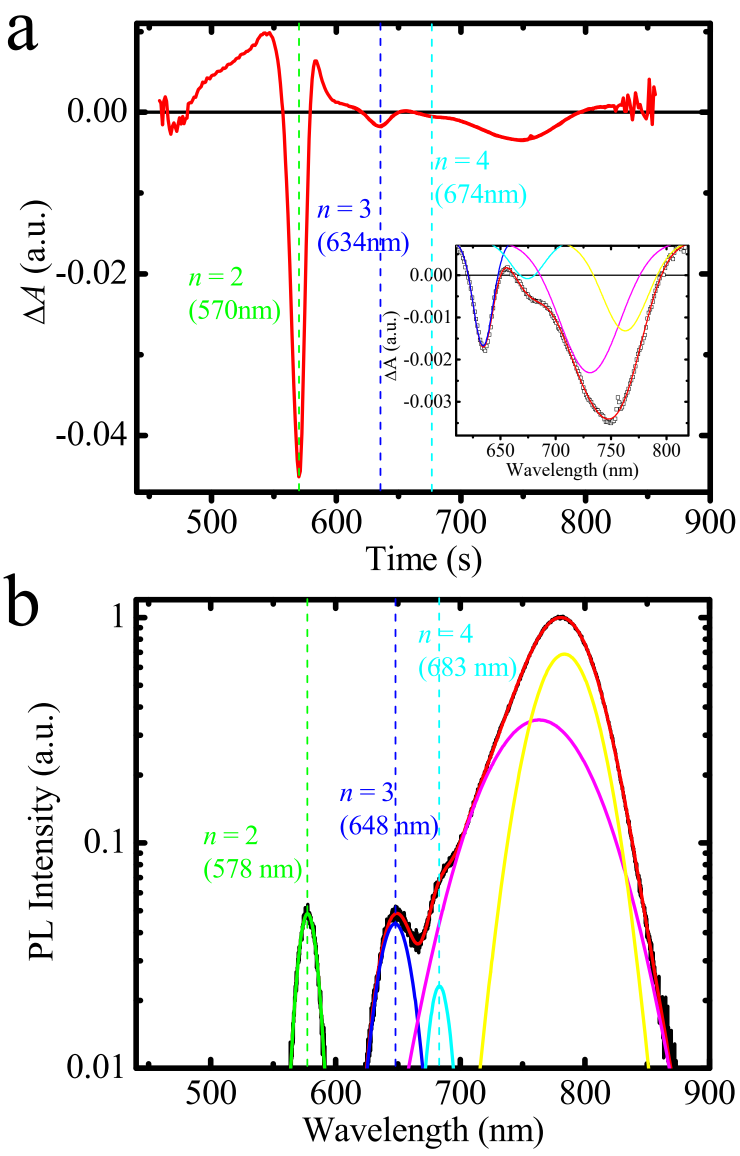

Supplementary Figure 10 | Exciton resonant peaks in the multi-quantum wells (MQWs). (a) Time delayed (100 ps) transient absorption (TA) spectrum for the perovskite MQWs film at a relatively high pump fluence of around 10 μJcm^{-2} (400 nm, 1 KHz, 100 fs). The inset shows the detailed Gaussian peak fittings. **(b)** Photoluminescence (PL) spectrum with Gaussian peak fittings for the perovskite MQWs at pump fluence of around 0.2 μJcm^{-2} (400 nm, 1 KHz, 100 fs).

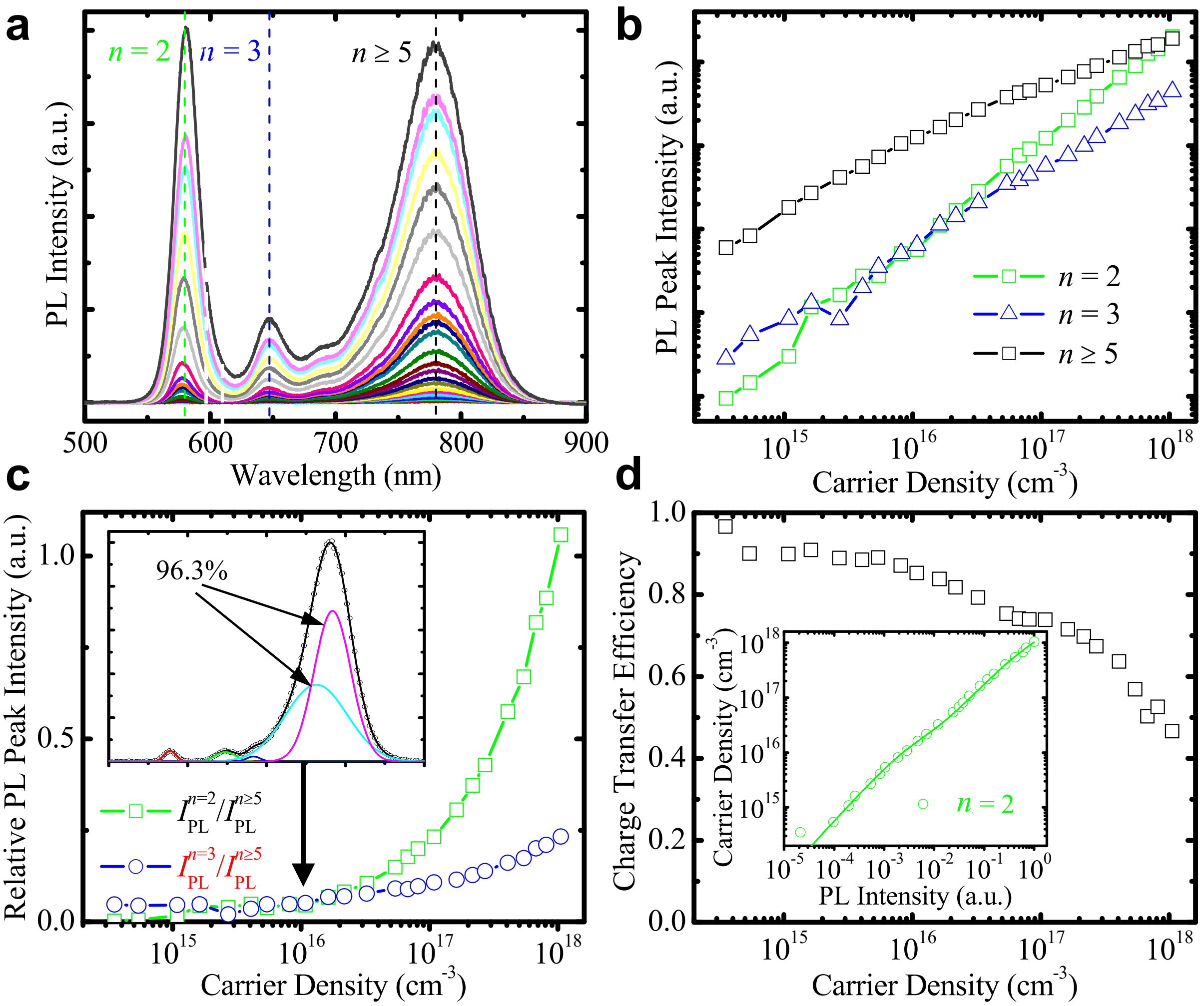

Supplementary Figure 11 | Carrier density dependent exciton localization from thin ($n \leq 4$) quantum wells (QWs) to thick ($n \geq 5$) QWs (TQWs). (a) Pump fluence dependent photoluminescence (PL) of the MQWs film. **(b)** The dependence of PL peak intensity on photon-injected carrier density at the exciton resonance of the QWs with different inorganic layers. **(c)** The injected carrier density dependent PL peak intensity ratios of bilayer and trilayer QW to TQW ($n \geq 5$). The inset shows the PL spectrum could be well fitted with 5 Gaussian peaks. The two dominant broad peaks are attributed to emission from the TQWs ($n \geq 5$), which occupy 96.3% of the total emission intensity at carrier density of around 1.0×10^{16}cm^{-3}. **(d)** The extracted injected carrier density dependence of carrier transfer efficiency from thin QW to TQW. The inset shows detailed fitting of the dependence of integrated PL intensity on the injected carrier density.

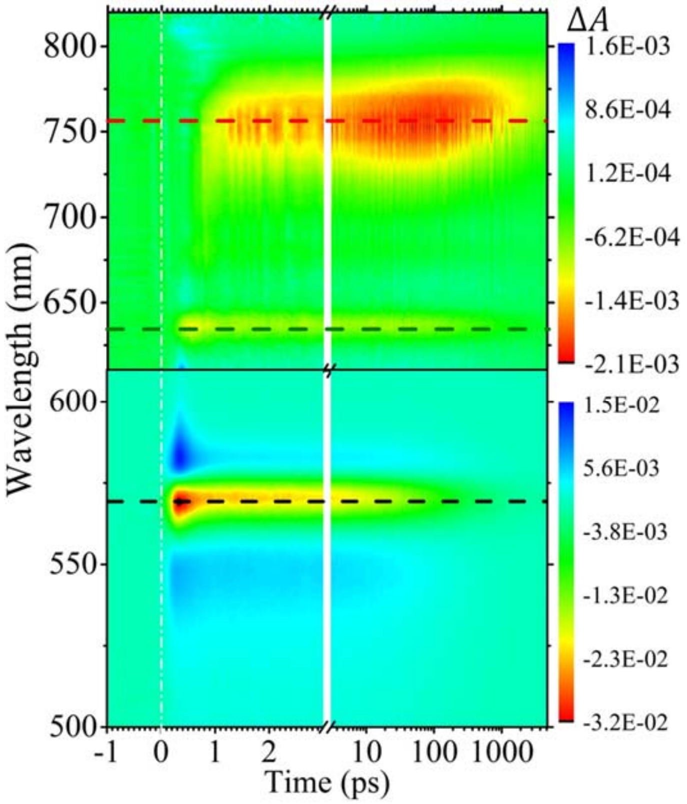

Supplementary Figure 12 | Probing exciton localization from thin ($n \leq 4$) quantum wells (QWs) to thick ($n \geq 5$) QWs (TQWs) with transient absorption (TA) method. TA spectra of the multi-QWs (MQWs) film following excitation at 400 nm (1 KHz, 100 fs, around 0.4 μJcm^{-2}). The color scale, vertical axis, and horizontal axis represent the photo-induced absorption changes (**ΔA)**, the probe wavelength, and the pump-probe time delay, respectively.

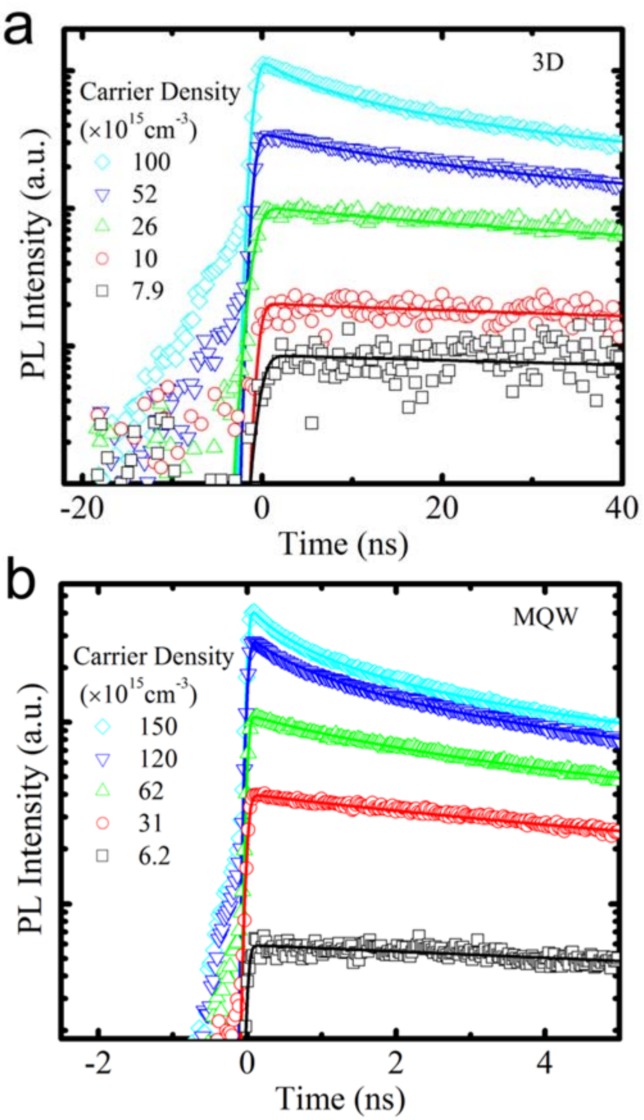

Supplementary Figure 13 | Measurement of the different order decay parameters. Time-resolved photoluminescence (TRPL) transients monitored over a certain time window for the three-dimensional (3D) perovskite **(a)** and perovskite multi-quantum wells (MQWs) **(b)** with a range of pulse fluences following excitation at 650 nm (1 KHz, 100 fs). The solid lines are global fittings with equation (1).

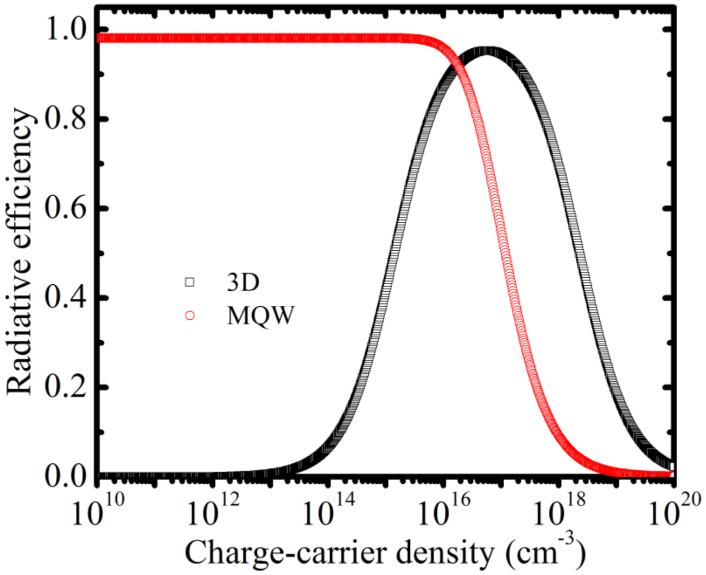

Supplementary Figure 14 | The predicted luminescence efficiency. The calculated radiative efficiencies for three-dimensional (3D) perovskite and perovskite multi-quantum wells (MQWs) with decay parameters extracted from the global fitting of time-resolved photoluminescence (TRPL) transients.

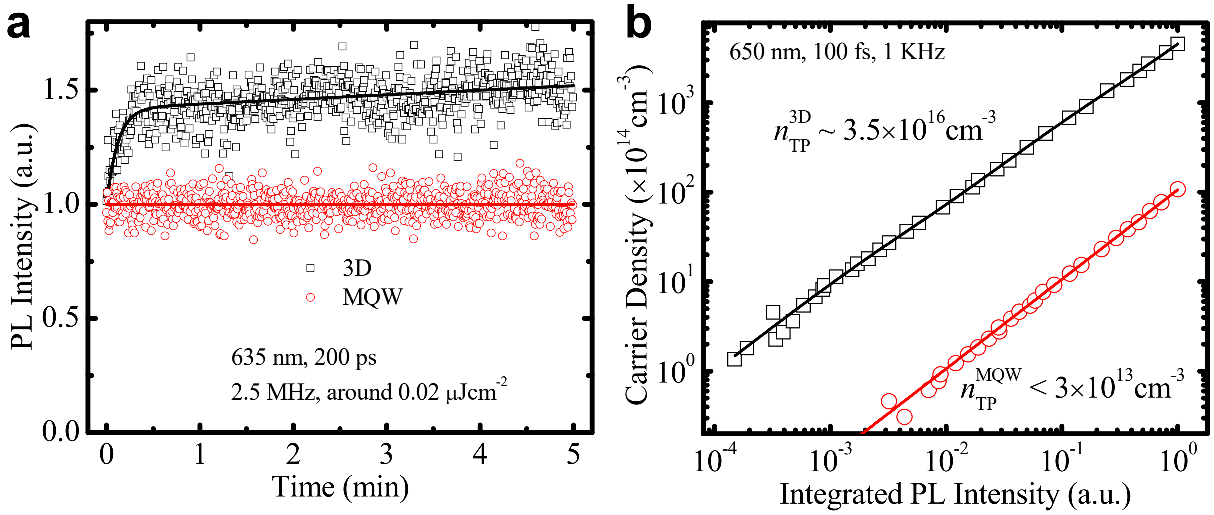

Supplementary Figure 15 | Probing trap states density in the perovskite films. (a) Light soaking induced integrated photoluminescence (PL) relative intensity change for thick ($n \geq 5$) quantum wells (TQWs) in the self-assembled MQWs film as well as for three-dimensional (3D) perovskite. The greatly increased PL intensity indicates part of the trap states in 3D perovskite were passivated with light induced re-crystallization, while the near invariant PL intensity indicates that the trap state density in TQWs is negligible. **(b)** PL intensity as a function of photon-generated exciton density within the low pump fluence range. The trap states density can be extracted by fitting the dependence with Supplementary equation 11.

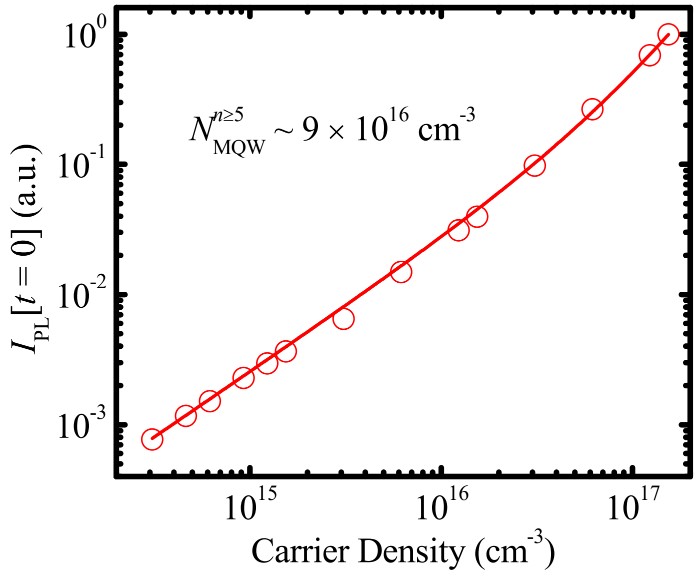

Supplementary Figure 16 | Determination of the thick ($n \geq 5$) quantum wells (TQWs) **doping concentration in the self-assembled multi-QWs (MQWs) film.** Photon-injected carrier density dependence of the initial time photoluminescence (PL) intensity ($I_{PL}(0)$) following excitation at 650 nm (1 KHz, 100 fs). The experimental data can be well-fitted (R^2 = 0.9997) with Supplementary equation 12 with a TQWs doping concentration of around 9×10^{16}cm^{-3}.

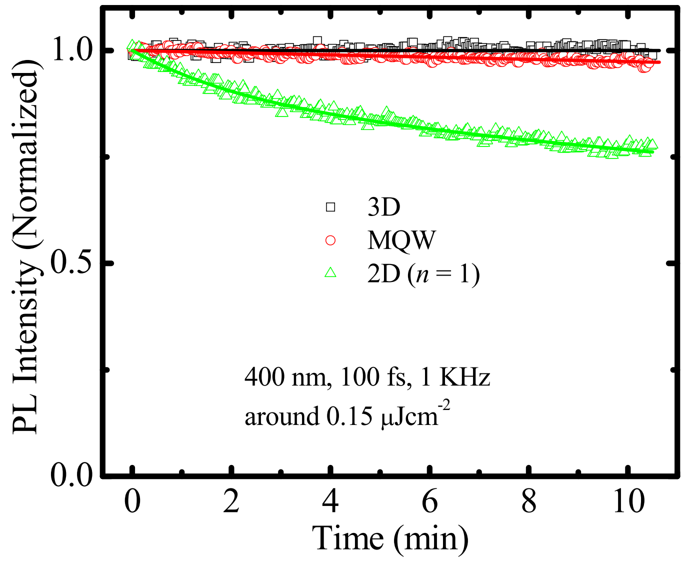

Supplementary Figure 17 | Photo-stability of the perovskite films under ultraviolet (UV) light excitation. The integrated photoluminescence (PL) intensity as a function of photon excitation time for the three-dimensional (3D), 2D (n = 1) and multi-quantum well (MQW) perovskite films.

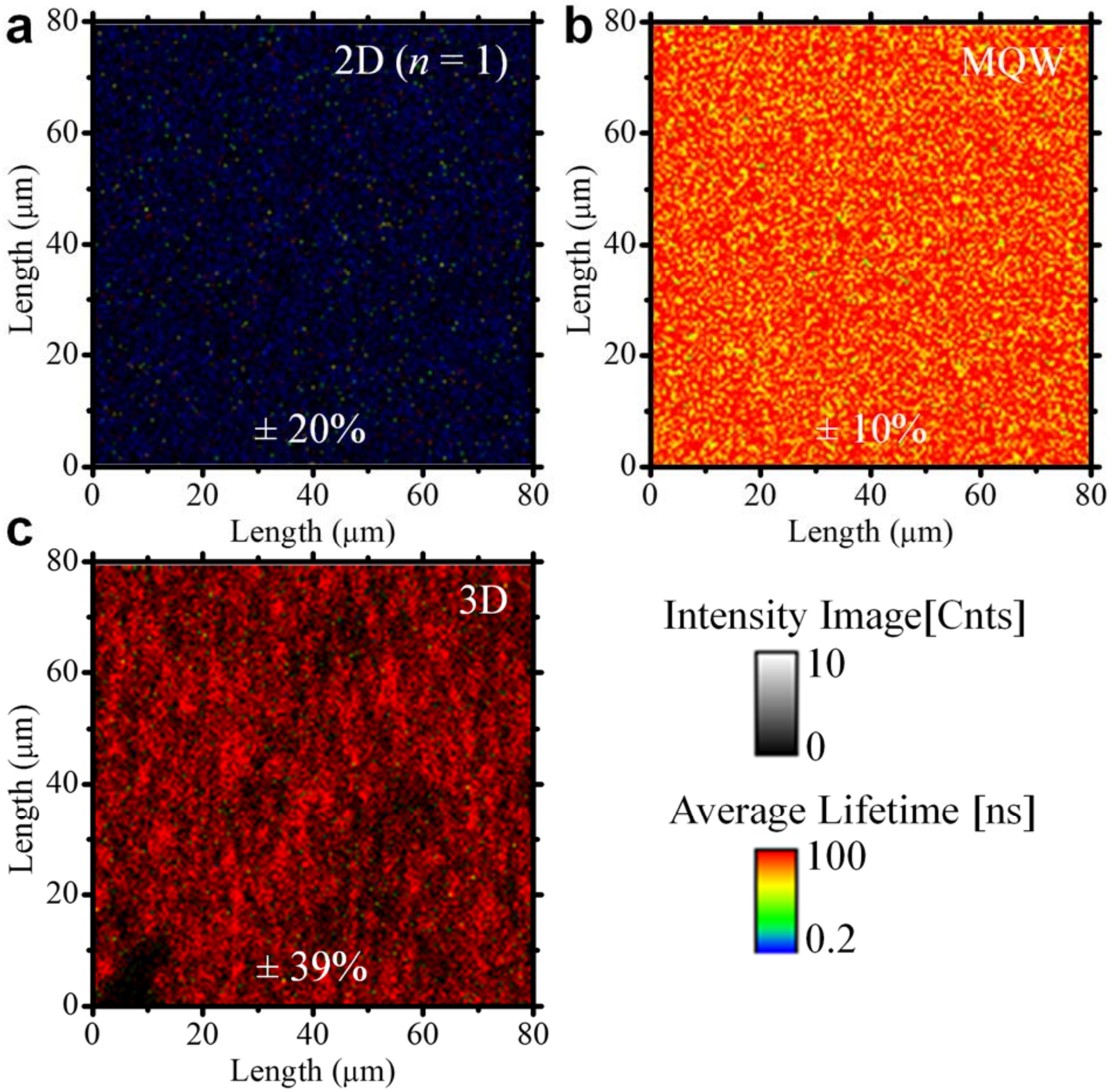

Supplementary Figure 18 | Photoluminescence (PL) intensity and lifetime imaging of the perovskite films. (a) – (c) PL imaging in counts and average lifetime for the single layer two-dimensional (2D) perovskite **(a)**, self-assembled perovskite multi-quantum wells (MQWs) **(b)**, and 3D perovskite **(c)**. The corresponding PL intensity standard deviations were taken over the scanned area (80×80 μm^2). The films were excited with 405 nm laser pulses (2.5 MHz, 200 ps, around 0.017 μJcm^{-2}) under the same experimental conditions, the emission was collected with a 490 nm long pass filter.

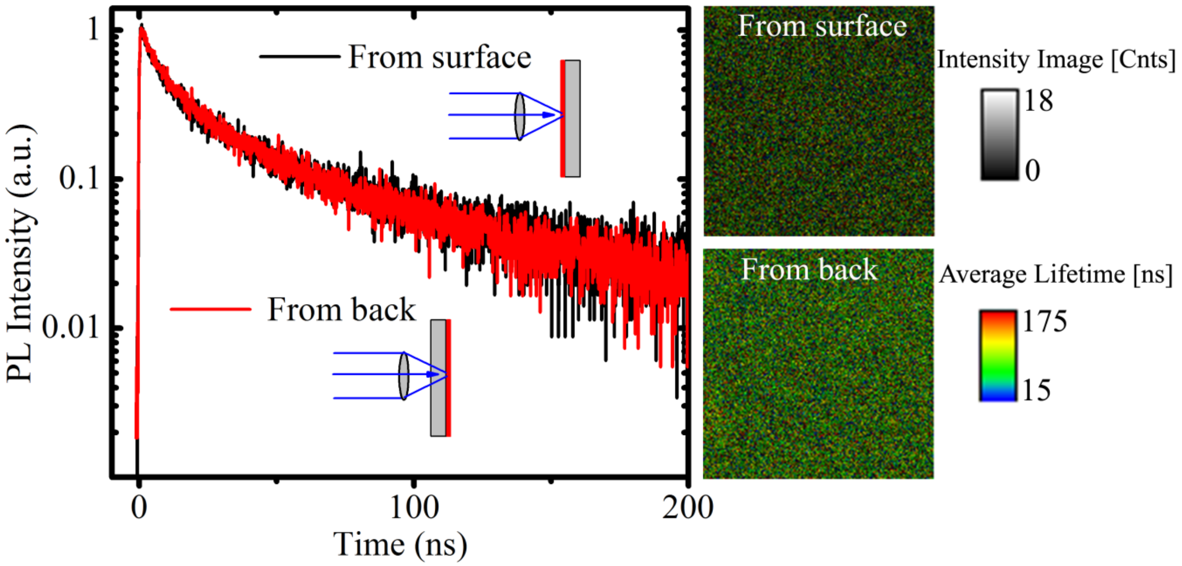

Supplementary Figure 19 | Determination of composition uniformity of the perovskite multi-quantum wells (MQWs) film across Z-axis. The identical photoluminescence (PL) decay transients collected from the film surface and back by a confocal microscopy suggest that there is no obvious composition gradient in the Z-axis. The films were scanned over 80×80 μm^2 with 405 nm laser pulses (2.5 MHz, 200 ps), the emission was collected with a 490 nm long pass filter.

Supplementary References

1. Hong, X., Ishihara, T., Nurmikko, A. V. Photoconductivity and electroluminescence in lead iodide based natural quantum-well structures. *Solid State Commun.* **84,** 657–661 (1992).
2. Era, M., Morimoto, S., Tsutsui, T., Saito, S. Organic-inorganic heterostructure electroluminescent device using a layered perovskite semiconductor $(C_6H_5C_2H_4NH_3)_2PbI_4$. *Appl. Phys. Lett.* **65,** 676–678 (1994).
3. Dou, L. *et al.* Atomically thin two-dimensional organic-inorganic hybrid perovskites. *Science* **349**, 1518-1521 (2015).
4. Tanaka, K., Kondo, T. Bandgap and exciton binding energies in lead-iodide-based natural quantum-well crystals. *Sci. Tecnol. Adv. Mater.* **4**, 599-604 (2003).
5. Hong, X., Ishihara, T., Nurmikko, A. V. Dielectric confinement effect on excitons in PbI4-based layered semiconductors. *Phys. Rev. B* **45**, 6961-6964 (1992).
6. Gauthron, K. *et al.* Optical spectroscopy of two-dimensional layered (C6H5C2H4-NH3)2-PbI4 perovskite. *Opt. Express* **18**, 5912-5919 (2010).
7. Calabrese, J. *et al.* Preparation and characterization of layered lead halide compounds. *J. Am. Chem. Soc.* **113,** 2328–2330 (1991).
8. Mitzi, D. B., Chondroudis, K., Kagan, C. R. Organic-inorganic electronics. *IBM J. Res. Dev.* **45,** 29–45 (2001).
9. Dohner, E. R., Jaffe, A., Bradshaw, L. R., Karunadasa, H. I. Intrinsic white-light emission from layered hybrid perovskites. *J. Am. Chem. Soc.* **136**, 13154-13157 (2014).

10. Xing, G. *et al*. Long-range balanced electron- and hole-transport lengths in organic-inorganic $CH_3NH_3PbI_3$. *Science* **342,** 344-347 (2013).
11. Xing, G., Luo, J., Li, H., Wu, B., Liu, X., Huan, C. H. A., Fan, H. J., Sum, T. C. Ultrafast exciton dynamics and two-photon pumped lasing from ZnSe nanowires. *Adv. Opt. Mater.* **1**, 319-326 (2013).
12. Landolt-Bornstein, Physics of II-VI and I-VII compounds, vol 17b, New Series, ed by O. Madelung (Springer, Berlin Heidelberg 1982).
13. Rudin, S., Reinecke, T. L., Segall, B. Temperature-depdendent exciton linewidths in semiconductors. *Phys. Rev. B* **42**, 11218-11231 (1990).
14. Wei, G., et al. Valley polarization in size-tunable monolayer semiconductor quantum dots. arXiv:1510.09135v1 (2015).
15. Wu, K., Bera, A., Ma, C., Du, Y., Yang, Y., Li, L., Wu, T. Temperature-dependent excitonic photoluminescence of hybrid organometal halide perovskite films. *Phys. Chem. Chem. Phys.* **16**, 22476-22481 (2014).
16. Wright, A. D. *et al.* Electron-phonon coupling in hybrid lead halide perovskites. *Nat. Commun.* **7**, 11755 (2016).
17. Cingolani, A., Ferrara, M., Lugara, M. Many-body effects in the emission spectrum of ZnTe under high-intensity photoexcitation. *Phys. Rev. B* **19**, 4149-4153 (1979).
18. Amani, M., *et al*. Recombination kinetics and effects of superacid treatment in Sulfur- and Selenium-based transition metal dichalcogenides. *Nano Lett.* **16**, 2786-2791 (2016).
19. Wehrenfennig, C., Eperon, G. E., Johnston, M. B., Snaith, H. J., Herz, L. M. High Charge Carrier Mobilities and Lifetimes in Organolead Trihalide Perovskites. *Adv. Mater.* **26**, 1584-1589 (2014).
20. Savenije, T. J., *et al.* Thermally activated exciton dissociation and recombination control the carrier dynamics in organometal halide perovskite. *J. Phys. Chem. Lett.* **5**, 2189-2194 (2014).
21. Saba, M., *et al*. Correlated electron-hole plasma in organometal perovskites. *Nat. Commun.* **5,** 5049 (2014).
22. Wehrenfennig, C., Liu, M. Z., Snaith, H. J., Johnston, M. B., Herz, L. M. Charge-carrier dynamics in vapour-deposited films of the organolead halide perovskite $CH_3NH_3PbI_{3-x}Cl_x$. *Energy Environ. Sci.* **7**, 2269-2275 (2014).
23. Yamada, Y., Nakamura, T., Endo, M., Wakamiya A., Kanemitsu, Y. Photocarrier Recombination Dynamics in Perovskite $CH_3NH_3PbI_3$ for Solar Cell Applications. *J. Am. Chem. Soc.* **136**, 11610-11613 (2014).
24. Unger, E. L., *et al*. Hysteresis and transient behavior in current-voltage measurements of hybrid-perovskite absorber solar cells. *Energy Environ. Sci.* **7**, 3690-3698 (2014).
25. Xing, G. *et al*. Low-temperature solution-processed wavelength-tunable perovskites for lasing. *Nat. Mater.* **13**, 476-480 (2014).
